# Metagenomics analysis revealed the distinctive ruminal microbiome and resistive profiles in dairy buffaloes

**DOI:** 10.1186/s42523-021-00103-6

**Published:** 2021-07-01

**Authors:** Hui-Zeng Sun, Ke-Lan Peng, Ming-Yuan Xue, Jian-Xin Liu

**Affiliations:** Institute of Dairy Science, Ministry of Education Key Laboratory of Molecular Animal Nutrition, College of Animal Sciences, Zhejiang University, Hangzhou, 310058 China

**Keywords:** Dairy buffaloes, Dairy cattle, Rumen, Antimicrobial resistant genes, Microbiome

## Abstract

**Background:**

Antimicrobial resistance poses super challenges in both human health and livestock production. Rumen microbiota is a large reservoir of antibiotic resistance genes (ARGs), which show significant varations in different host species and lifestyles. To compare the microbiome and resistome between dairy cows and dairy buffaloes, the microbial composition, functions and harbored ARGs of rumen microbiota were explored between 16 dairy cows (3.93 ± 1.34 years old) and 15 dairy buffaloes (4.80 ± 3.49 years old) using metagenomics.

**Results:**

Dairy buffaloes showed significantly different bacterial species (LDA > 3.5 & *P* < 0.01), enriched KEGG pathways and CAZymes encoded genes (FDR < 0.01 & Fold Change > 2) in the rumen compared with dairy cows. Distinct resistive profiles were identified between dairy cows and dairy buffaloes. Among the total 505 ARGs discovered in the resistome of dairy cows and dairy buffaloes, 18 ARGs conferring resistance to 16 antibiotic classes were uniquely detected in dairy buffaloes. Gene *tcmA* (resistance to tetracenomycin C) presented high prevalence and age effect in dairy buffaloes, and was also highly positively correlated with 93 co-expressed ARGs in the rumen (*R* = 0.98 & *P* = 5E-11). In addition, 44 bacterial species under *Lactobacillus* genus were found to be associated with *tcmA* (*R* > 0.95 & *P* < 0.001). *L. amylovorus* and *L. acidophilus* showed greatest potential of harboring *tcmA* based on co-occurrence analysis and *tcmA*-containing contigs taxonomic alignment.

**Conclusions:**

The current study revealed distinctive microbiome and unique ARGs in dairy buffaloes compared to dairy cattle. Our results provide novel understanding on the microbiome and resistome of dairy buffaloes, the unique ARGs and associated bacteria will help develop strategies to prevent the transmission of ARGs.

**Supplementary Information:**

The online version contains supplementary material available at 10.1186/s42523-021-00103-6.

## Background

Antimicrobial resistance (AMR) is one of the biggest threats to human health and causes several to ten million death annually in the world [1]. The wide spread of antimicrobial resistance genes (ARGs), especially for those that confer resistance to clinical relevance or “last defense line” antibiotics, requires urgent tasks to reveal the harbor sources and transmission routes of diverse ARGs [2]. The microbiome of livestock gastrointestinal tracts serves as important reservoirs for ARGs [3–6], which is largely attributed to the long-term use of antibiotic in veterinary medicine or sub-therapeutic doses of antibiotic supplied in the feed acting as growth promoter [7]. The microbial ARGs are mainly structured by the bacterial phylogeny [8]. The ruminal bacterial composition and diversity are much higher than fecal microbiome in dairy cows [9], suggesting that rumen microbiome is a larger reservoir of ARGs.

The ARGs harbored in direct-fed microbials or silage inoculants that isolated from rumen microbiome (e.g. lactic acid bacteria or fibrolytic bacteria) [10] could be directly or indirectly transmitted and accumulated in human and other food animals. In addition, the bacterial ARGs in the rumen will easily spread to the environment, such as soil and water, through rumination and saliva, which will lead to drug-selective pressure on environmental and human life related bacterial communities [11]. The high-throughput metagenomics technology has been gradually applied into functional annotation and ARGs identification of large amounts of uncultured bacteria in gut microbiome of different species [12]. Nevertheless, the ruminal resistome information is still limited [13], especially for more diverse ruminant species.

As an important ruminant species, buffaloes are estimated to be more than 200 million worldwide and made great contribution to the nutrition and health of humankind [14]. Compared with dairy cows, dairy buffaloes have some unique physiological patterns, such as higher nutritious milk for cheese and cream production [15], better adaptability to low-quality and less-digestible roughage, hot and humid climate [16], advantages in resistance and susceptibility to common bovine disease [17]. Comparative analysis of rumen microbiome nowadays attracts increasing interest to understand the above distinct physiological patterns in dairy buffaloes [18].

Dairy buffaloes are generally raised in a low-density system with waterlogged conditions [19], which exhibit close interactions with the environment. Therefore, it is hypothesized that more diverse and distinctive microbiome and resistome existed in the rumen of dairy buffaloes compared with dairy cows. Here, we collected rumen samples from 16 dairy cows and 15 dairy buffaloes to perform compositional and functional comparative analysis using metagenomics. Since the average culling time for dairy buffaloes worldwide is generally longer than that of dairy cows (> 10 vs. 5 ~ 6 years old), dairy buffaloes with different ages (from 1 to 10 years old) were selected to reveal the comprehensive rumen microbial profiles. The current study provides comprehensive understanding on microbiome and resistome profiles of dairy buffaloes, and uncovers potential risks to clinical treatment and human health caused by spreading ruminal ARGs from dairy buffaloes.

## Methods

### Experimental design, animals and sample collection

All experimental procedures were approved by the Animal Care Committee of Zhejiang University (Hangzhou, China). A total of 16 dairy cows and 15 dairy buffaloes were used in this study. Sixteen Holstein dairy cows (3–5 year-old) were selected from a commercial farm in Hangzhou (China). Fifteen Murrah dairy buffaloes with different ages (Y: 12 month-old, *n* = 5; M: 3–5 year-old, *n* = 4; E: 6–8 year-old, *n* = 3; O: > 9-year-old, *n* = 3) were selected from buffalo farm of Buffalo Research Institute, Chinese Academy of Agricultural Sciences (Nanning, China). The animals were healthy and received no therapeutic or prophylactic antimicrobial treatment during the current lactation cycle or in the past year. All the selected animals were healthy and received no therapeutic or prophylactic antimicrobial treatment based on the veterinary records. The diet of dairy cows and dairy buffaloes were supplemented (Table S1). Fifteen mL of rumen fluid were collected from each animal before morning feeding using an oral stomach tube as described previously [20]. The first 50–100 mL of rumen fluids in each samping were discarded to remove the potential saliva contamination. The rumen content samples were immediately frozen in liquid nitrogen and stored at − 80 °C until further analysis.

### DNA extraction, library construction, sequencing and data processing

Total DNA of rumen contents (3 mL) was extracted using the repeat bead-beating plus column method [21]. The concentration and quality of DNA were evaluated using a NanoDrop 2000 spectrophotometer (NanoDrop Technologies, Wilmington, DE, USA) and 1% agarose gel. The Covaris M220 (Covaris lnc., Woburn, MA, USA) was used to generate 300 bp DNA fragments. The TrueSeq DNA PCR-Free Library Prep Kits (Illumina, San Diego, CA, USA) were applied to perform metagenome libraries construction according to the manufacturer’s protocol. Metagenomic sequencing (PE150) was performed on an Illumina HiSeq 3000 platform (Illumina, San Diego, CA, USA). Totally, 189.5 Gb clean data (from 194.5 Gb raw data) with an average of 61.11 million clean reads in each sample were generated.

The “N” records, 3′- and 5′-ends reads, short reads (< 50p) and low-quality bases (Phred score < 20) were filtered using fastp software [22]. Host genomic and adaptor contaminations were removed by mapping to the *Bos taurus* and *Bubalus bubalis* genomes using Burrows-Wheeler alignment (BWA) software [23]. After trimming, a set of high-quality and free of host genomic contaminants reads were obtained for further analysis.

The clean reads were de novo assembled for each sample using Megahit software based on succinct de Bruijn graph method [24]. The assembled contigs with the length greater than 300 bp were predicted to open reading frames using MetaGene software [25]. A total of 23,963,622 contigs with the length from 300 to 415,681 bp were obtained. Assembled contigs were pooled and then used to construct non-redundant (NR) gene catalog using CD-HIT with 90% identity and 90% coverage [26]. Gene abundances were calculated as gene reads/gene length using SOAPaligner software based on 95% identity [27]. And when comparing the taxa difference between two groups, the gene abundance was normalized as relative abundance: reads abundance of taxon A in sample 1/total reads abundances of all taxa in sample 1 × 100, to remove the bias of sequencing depth among different samples.

### Taxonomic classification and functional annotation

Taxonomic classification was performed using DIAMOND software [28] to map the sequence of NR gene catalog against the NR database (ftp://ftp.ncbi.nlm.nih.gov/blast/db/). Taxonomic abundance profiles were generated at domain, kingdom, phylum, class, order, family, genus and species levels. The relative abundance of each taxon was calculated and normalized using the proportion of each taxa identified in the same sample. Only the taxa with a relative abundance > 0.1 in more than 50% of animals within each group were selected for downstream analysis. Stacked histogram was used to display the abundance difference of phylum and family levels bacteria.

The sequence of NR gene catalog was mapped to Kyoto Encyclopedia of Genes and Genomes database (KEGG, http://www.genome.jp/kegg/) using Diamond with BLASTP (http://blast.ncbi.nlm.nih.gov/Blast.cgi) type and an e value of 1E-5. Pathway annotation was then conducted using KOBAS 2.0 according the blast results [29]. Carbohydrate-active enzymes were identified based on HMMscan tools against the Carbohydrate-Active enZYmes Database (CAZy) with hmmer type and e-value ≤1E-5 [30]. Abundances of KEGG pathway and CAZymes were normalized into counts per million reads (CPM) for further comparison analysis with the cutoff of CPM > 5 in greater than 50% of animals in at least one group.

### ARGs identification and resistome analysis

The sequence of NR gene catalog was mapped to the Comprehensive Antimicrobial Resistance Database (CARD) using Diamond software with BLASTP type and an e-value ≤1E-5 to identify the ARGs [28]. The corresponding AMR mechanisms and target antibiotics of ARGs were also provided in the CARD database [28]. The read counts of ARGs in each sample were normalized into CPM and only the ARGs with CPM value > 1 in more than 50% of animals in at least one group were used for down-stream analysis. Unique ARGs were defined as the genes absent in one group but with CPM values > 1 in all samples in the other group.

Supervised partial least-squares discrimination analysis (PLS-DA) was performed using ropls package [31] in R software to reveal discriminate patterns of ARGs between dairy cow and dairy buffalo groups. Pheatmap package in R was used to analyze the classification of ARGs and samples based on ARGs expression abundance. The co-expressed ARGs modules and their complex correlations with unique ARGs in dairy buffaloes were characterized using weighted gene co-expression network analysis (WGCNA) package in R [32]. To achieve a signed R^2^ value of scale-free topology model greater than 0.9 [33], the soft-thresholding power value was set as 9. The minimum count of genes assigned in each module was selected at 10. The cut-off of significantly correlated gene modules was defined as module-unique ARGs relationship |R| > 0.7 & *P* < 0.01. The association between membership and gene significance in the most correlated module was further explored. The correlation between the *tcmA* and bacterial species in the rumen of dairy buffaloes were calculated by Hmisc package in R. The co-occurence network was visualized by Cytoscape software [34].

### Statistical analysis

The Bray-Curtis dissimilarity matrices based principal-coordinate analysis (PCoA) and *P* values calculation at genus and species level were performed using adae4 (version 1.7.13) [35] and vegan (version 2.5.4) [36] packages in R software. The significantly different features (SDFs) at each taxa level between dairy cows and dairy buffaloes were analyzed using least discriminant analysis (LDA) Effect Size (LEfSe) [37], in which both statistical significance and biological relevance were considered. The SDFs was defined at the thresholds of *P* < 0.01 & LDA > 3.5. The KEGG pathways and CAZymes between dairy cow and dairy buffalo groups were compared using the Wilcoxon rank-sum test and displayed in the volcano plot using R. The functional memberships with a false discovery rate (FDR) < 0.01 & |fold change (FC)| > 2 were considered as significantly different. The extremely higher CAZymes were defined as FDR < 0.01 & FC > 25. The difference of unique ARGs abundance among Y, M, E and O groups in dairy buffaloes were analyzed using ANOVA test with a *P* < 0.05 considered as statistical significance.

## Results

### Different rumen bacterial taxonomic compositions between dairy cows and dairy buffaloes

**Fig. 1:**
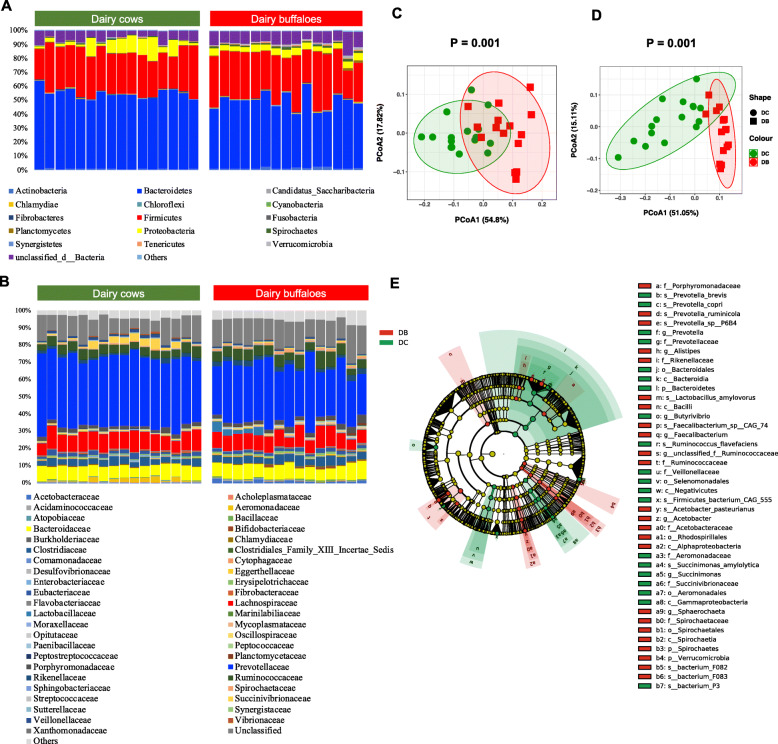
Rumen bacterial compositional profiles of dairy cows and dairy buffaloes. **A** Relative abundance of major bacteria phyla (relative abundance > 0.1% in more than 50% animals) for all individuals. **B** Relative abundance of major bacteria families (relative abundance > 0.1% in more than 50% animals) for all individuals. **C** Principal coordinate analysis (PCoA) of bacteria genera based on Bray-Curtis distance. **D** PCoA of bacteria species based on Bray-Curtis distance. **E** Cladogram of significantly different taxa identified in the rumen microbiome data sets of dairy cows and dairy buffaloes based on the cut-off of LDA > 3.5 and *P* < 0.01. Clades significantly enriched in each cohort are highlighted by the colors shown in the legend. DB: dairy buffalo; SDF: significantly different feature; LDA: linear discriminant analysis

Bacteria was most predominant in all animals (Table S2) and showed significantly higher relative abundance in dairy buffaloes than in dairy cows (96.56% vs. 94.25%, *P* < 0.001). At phylum and family levels, dairy buffaloes had similar bacterial composition with dairy cows (Fig. 1A and B**)**. Totally, 15 different bacterial phyla were identified (relative abundance > 0.1% in more than 50% animals), with *Bacteroidetes*, *Firmicutes, Proteobacteria, Actinobacteria* and *Spirochaetes* being the top 5 most abundant (accounting for > 89% of totally bacterial community) phyla in both dairy cows and dairy buffaloes (Fig. 1A). At family level, 45 different bacterial taxa were identified, with *Prevotellaceae*, *Lachnospiraceae*, *Bacteroidaceae*, *Ruminococcaceae* and *Clostridiaceae* being the top 5 most abundant (accounting for > 60% of totally bacterial community) families in both dairy cows and dairy buffaloes (Fig. 1B). At genus and species levels, the bacterial community displayed significantly different profiles between dairy cows and dairy buffaloes in PCoA plot (*P* = 0.001, Fig. 1C and D). Among the 44 SDFs under the cutoff of LDA > 3.5 and *P* < 0.01, 24 bacterial taxa were significantly higher, while 20 bacterial taxa were significantly lower in dairy buffaloes than dairy cows (Fig. 1E). The key species significantly enriched in the rumen of dairy buffaloes including *Bacterium F082*, *Prevotella ruminicola*, *Faecalibacterium sp. CAG:74*, *Lactobacillus amylovorus*, *Prevotella sp. P6B4*, *Acetobacter pasteurianus*, and *Bacterium F083* (Fig. 1E**)**.

### Functional difference of rumen microbiome between dairy cows and dairy buffaloes

**Fig. 2:**
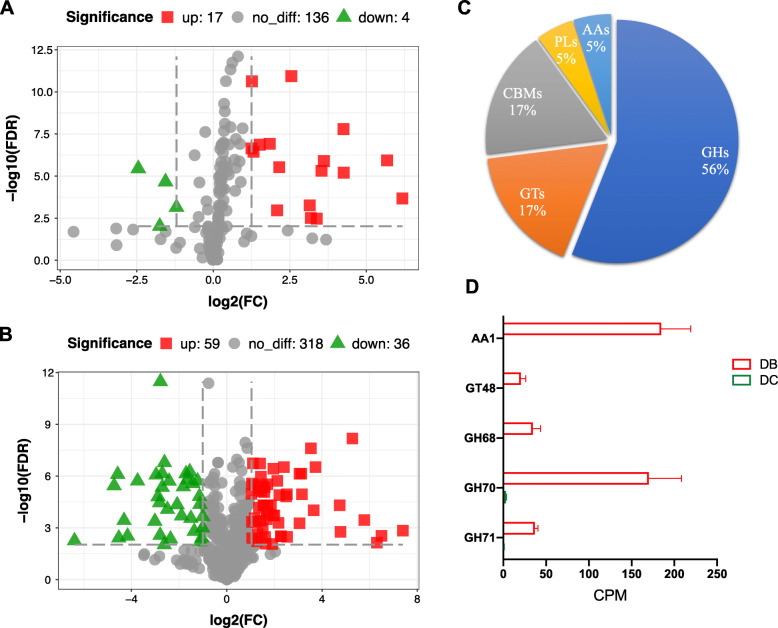
Functional difference of ruminal microbiome between dairy cows and dairy buffaloes. **A** The volcano plot of significantly different 3rd level KEGG pathways between dairy cows and dairy buffaloes. **B** The volcano plot of significantly different CAZymes between dairy cows and dairy buffaloes. **C** The distribution of significantly different CAZymes families between dairy cows and dairy buffaloes. **D** The relative abundance of extremely higher CAZymes encoded genes in the dairy buffaloes. FC: fold change (dairy buffaloes/dairy cows); GHs: glycoside hydrolases; PLs: polysaccharide lyases; CBMs: carbohydrate-binding modules; GTs: glycosyltransferase; AAs: auxiliary activities; DB: dairy buffalo; DC: dairy cow; CPM: counts per million

By aligning the NR gene catalog to the KEGG and CAZy database, metabolic pathways and carbohydrate-active enzymes were analyzed and compared between dairy cows and dairy buffaloes. Among the 21 significantly different third-level pathways between two groups (FDR < 0.01 & |FC| > 2), 17 pathways were enriched in the dairy buffaloes and 4 pathway were enriched in the dairy cows (Fig. 2A).

For the CAZymes profiles, a total of 413 genes encoding family-level CAZymes were identified in the rumen microbiome of dairy cows and dairy buffaloes (Table S3). As shown in Fig. 2B, 59 of CAZymes encoded genes were up-regulated and 36 were down-regulated in dairy buffaloes than in dairy cows (FDR < 0.01 & |FC| > 2). Among the CAZymes encoded genes significantly higher expressed in the dairy buffaloes (Fig. 2C), 56% of them belonged to glycoside hydrolases (GHs), 17% to glycosyltransferase (GTs), 17% to carbohydrate-binding modules (CBMs), 5% to polysaccharide lyases (PLs), and 5% to auxiliary activities (AAs). The extremely higher CAZymes encoded genes in dairy buffaloes were GH71 (FDR = 6.70E-9, FC = 38.72), GH70 (FDR = 3.49E-4, FC = 54.86), GH68 (FDR = 0.0015, FC = 167.7), GT48 (FDR = 0.003, FC = 90.53), AA1 (FDR = 4.89E-05, FC = 26.77) (Fig. 2D).

### Resistome profiles of the rumen microbiome and distinct ARGs patterns between dairy cows and dairy buffaloes

**Fig. 3:**
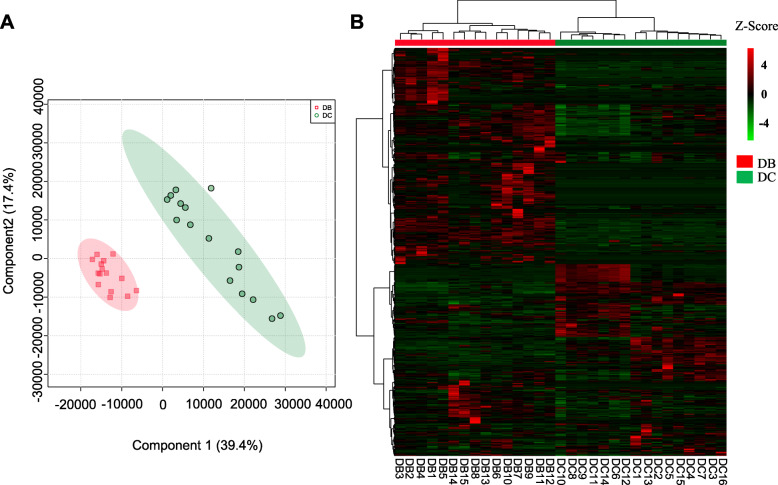
Rumen resistome profiles in dairy cows and dairy buffaloes. **A** The principal component analysis of ARGs identified in the rumen of dairy cows and dairy buffaloes. **B** The heatmap of the relative abundance of all the ARGs in all the individuals

Dairy buffaloes showed distinct resistome profiles from dairy cows (Fig. 3). Totally, 505 ARGs were detected in rumen microbiome of dairy cows and dairy buffaloes; and 435 of them were expressed in at least one group (CPM > 1 in more than 50% animals). In the PLS-DA plot, the resistome profiles displayed clearly discrimination between dairy cows and dairy buffaloes, in which the PC1 explained 39.4% of total variations (Fig. 3A). The heatmap revealed that 16 dairy cows and 15 dairy buffaloes were assigned into two separated groups (Fig. 3B). More than 60% of ARGs showed higher expression level in dairy buffaloes than in dairy cows in the heatmap overview (Fig. 3B). The relative abundance of top 10 most abundant ARGs accounted for about 32.10 and 33.13% of total reads in dairy cows and dairy buffaloes, respectively (Table S4). *Staphylococcus aureus rpoC conferring resistance to daptomycin* was the most abundant ARG in both groups. In terms of AMR phenotypes of ruminal resistome in dairy buffaloes, 33.13% of the sequence reads corresponded to fluoroquinolone (11.87%), lipopeptide antibiotic (7.46%), and aminocoumarin (6.20%) AMR (Table S5).

### Unique ruminal ARGs in dairy buffaloes and their resistance mechanisms

**Fig. 4:**
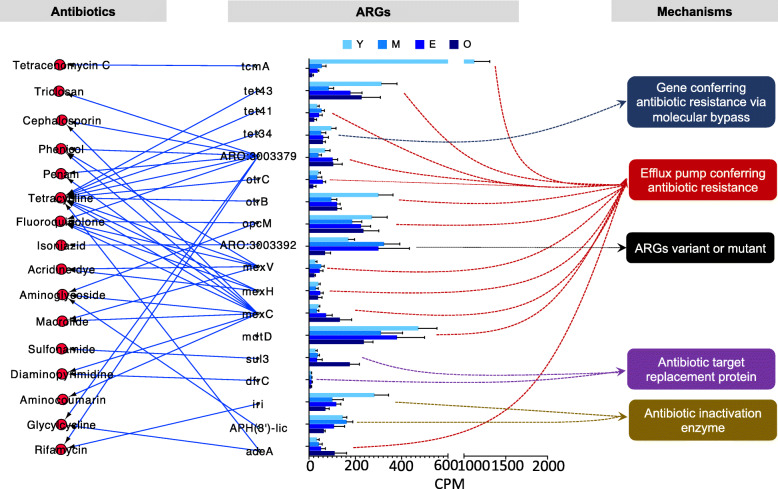
The unique ruminal ARGs detected in the dairy buffaloes. The relative abundance, target antibiotics and resistant mechanisms of 18 unique ARGs identified in each sub-group (Y/M/E/O). ARGs: antibiotic resistance genes

Eighteen ARGs were uniquely detected in the rumen microbiome of dairy buffaloes (CPM > 1 in all samples of dairy buffaloes and CPM = 0 in all samples of dairy cows) (Fig. 4). These ARGs included *tcmA* (CPM = 399.92), *tet43* (CPM = 208.52), *tet41* (CPM = 39.44), *tet34* (CPM = 70.76), *ARO:3003379* (CPM = 75.58), *otrC* (CPM = 38.84), *otrB* (CPM = 174.49), *opcM* (CPM = 232.42), *ARO:3003392* (CPM = 216.70), *mexV* (CPM = 37.12), *mexH* (CPM = 39.07), *mexC* (CPM = 64.17), *mdtD* (CPM = 363.64), *sul3* (CPM = 61.65), *dfrC* (CPM = 9.93), *iri* (CPM = 158.81), *APH (3′)-IIc* (CPM = 126.53), and *adeA* (CPM = 55.19) (Table S6). Unique ruminal ARGs showed large individual variance within dairy buffaloes (Fig. 4). The relative abundance of *tcmA* (*P* = 0.002), *otrB* (*P* = 0.021), *sul3* (*P* = 0.043), *iri* (*P* = 0.021) presented significant difference among different age groups of dairy buffaloes (Fig. 4).

Most of the unique ARGs (*tcmA*, *tet43*, *tet41*, *ARO:3003379*, *otrC*, *otrB*, *opcM*, *mexV*, *mexH*, *mexC*, *mdtD*, *adeA*) conferred resistance through the mechanism of “efflux pump conferring antibiotic resistance” (Fig. 4). The resistance mechanisms of *tet34*, *ARO:3003392*, *sul3* and *dfrC*, *iri* and *APH (3′)-IIc* were “gene conferring antibiotic resistance via molecular bypass”, “ARGs variant or mutant”, “antibiotic target replacement protein”, and “antibiotic inactivation enzyme”, respectively (Fig. 4). These ARGs conferred resistance to tetracenomycin C, tetracycline, glycylcycline, aminoglycoside, rifamycin, diaminopyrimidine, sulfonamide, aminocoumarin, phenicol, fluoroquinolone, penam, macrolide, cephalosporin, acridine dye, isoniazid, and triclosan (Fig. 4).

### The relationship between unique ARGs and bacterial species in the rumen of dairy buffaloes

**Fig. 5:**
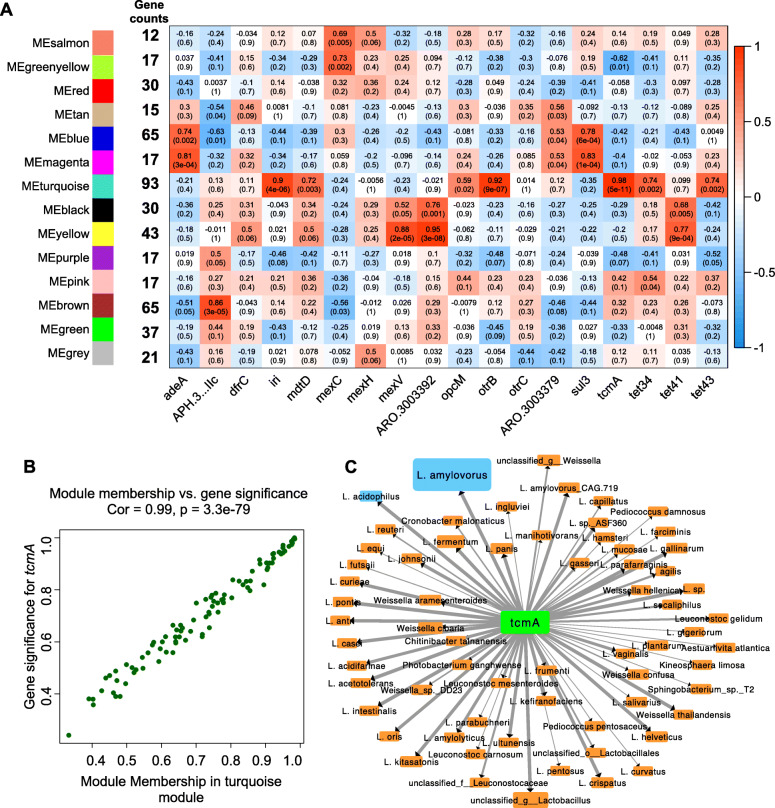
The relationship between unique ARGs and bacterial species in the rumen of dairy buffaloes. **A** The relationship between 18 unique ARGs and co-expressed ARGs in the rumen of dairy buffaloes. **B** The relationship between members in significantly correlated gene module and gene significance for *tcmA*. **C** The co-occurrence network of *tcmA* and its highly associated bacteria species in the rumen of dairy buffaloes

To investigate the roles of unique ARGs in the ruminal resistome of dairy buffaloes, the relationship between co-expressed ARGs modules and unique ARGs was analyzed using WGCNA. Fourteen co-expressed gene modules that contained different number of ARGs (12–93) were identified (Fig. 5A). Totally, 16 significantly correlated relationships between 7 gene modules and 18 unique ARGs were observed (|R| > 0.7 & *P* < 0.01). Among them, MEturquoise module was positively associated with 6 ARGs, including *tcmA* (*R* = 0.98 & *P* = 5E-11), *otrB* (*R* = 0.92 & *P* = 9E-07), *iri* (*R* = 0.90 & *P* = 4E-06), *tet34* (*R* = 0.74 & *P* = 0.002), *tet43* (*R* = 0.74 & *P* = 0.002), and *mdtD* (*R* = 0.72 & *P* = 0.003) (Fig. 5A). The co-expressed membership in MEturquoise module and gene significance for *tcmA* displayed highest relationship (*R* = 0.99, *P* = 3.3E-79) (Fig. 5B).

The co-occurrence patterns between bacterial species in the rumen of dairy buffaloes and *tcmA* gene were further explored using network analysis (Fig. 5C). Totally, 65 bacterial species were highly positively correlated (*R* > 0.95, *P* < 0.01) with *tcmA*, with 44 belonging to *Lactobacillus* genus (Fig. 5C). *L. amylovorus* (*R* = 0.993, *R* = 2.77 E-13) and *L. acidophilus* (*R* = 0.992, *R* = 4.94 E-13) showed strongest positive association with *tcmA* (Fig. 5C). It is notable that *L. amylovorus* (FC = 269.9, *P*_*adjust*_ < 0.001) and *L. acidophilus* (FC = 11.84, *P*_*adjust*_ < 0.001) were highly enriched in the rumen of dairy buffaloes than in dairy cows.

## Discussion

To our knowledge, this study should be one of the first study to compare rumen microbiome between dairy cows and buffaloes using metagenomics. Many factors can affect and shape the rumen microbiome, such as diet, breed, age, etc. It is reported that age plays important roles in rumen microbiome dynamics [38]. Dairy buffaloes with different ages (from 1 to 10 year old) was selected in this study to reveal the comprehensive profiles of rumen microbiome as well as to explore the age effects on resistome. Using q-PCR and 16S rRNA gene sequencing, it has been reported that *Bacteroidetes* (accounting for 42–72% of total bacteria) and *Prevotella* (accounting for 22–58% of total bacteria) were the most abundant phylum and genus bacteria in dairy buffaloes [39], which were consistent with our results (accounting for 37–60% and 22–44% of total bacteria). We also found that the top abundant bacterial phyla and families were consistent in dairy cows and dairy buffaloes. Even though dairy buffaloes showed distinct microbial community composition and higher Shannon diversity from dairy cows under the similar diet [18], the major rumen microbial profiles especially at phylum level show strong similarities between cattle and buffaloes [40, 41]. Lin et al. reported that no correlation between rumen microbiota and diet [39] while significant association between rumen microbiota composition and milking performance were observed [42] in dairy buffalo. It is indicated that dairy cows and dairy buffaloes shared some core rumen microbiota regardless of diet and geographical location, but the differed microbiota at higher taxonomic levels may reflect the breed or functional difference. We found the diet-induced SDFs were likely to be included in the comparisons between dairy cows and dairy buffaloes with relative lower strict thresholds, therefore, the SDFs revealed under the cutoff of LDA > 3.5 and *P*_*adjust*_ < 0.01 were further investigated.

*Prevotella ruminicola* was the most abundant bacterial species in our dataset in both dairy cows and buffaloes, consistent with previous reports [43, 44]. *Prevotella ruminicola* can efficiently utilize ammonia nitrogen and peptides for microbial protein synthesis [45]. The optimal adaptative mechanism of *Prevotella ruminicola* in the rumen environment makes it maximize nitrogen assimilation [46]. It is reported that the *Prevotella ruminicola* population increased with the increasing dietary crude protein (CP) levels [47, 48]. However, the dietary CP in dairy buffalo was lower than that in dairy cow (13.8% vs. 16.1%). The opposite results (the *Prevotella ruminicola* was significantly enriched in dairy buffaloes) suggest that *Prevotella ruminicola* abundance in the rumen was not only driven by dietary CP but may also be influenced by microbial-microbial interaction and host effect between dairy cows and buffaloes. Another significantly enriched *prevotella* species (*Prevotella sp. P6B4*) in dairy buffaloes was also abundant bacteria detected in rumen across the globe [49]. The two species were predicted to have 27 and 26 polysaccharide utilization locus [50], indicating that the rumen bacteria in dairy buffaloes have higher breakdown capacity of complex glycans than that in dairy cows. The higher abundance of *Acetobacter pasteurianus* and *Lactobacillus amylovorus* in the rumen contributed to the greater degradation ability of cellulose in dairy buffaloes. The function of *Faecalibacterium sp. CAG:74, Bacterium F082* and *Bacterium F083* species has not been well characterized, nevertheless, the high metabolic activity of these bacteria was approved [51]. The potential higher levels of metabolic function and carbohydrate degradation in the rumen microbiome of dairy buffaloes were supported by functional analysis.

More than 62% of significantly different metabolic pathways and CAZymes between two groups were enriched in dairy buffaloes, which provides further evidence that dairy buffaloes were enhanced in metabolism, especially for carbohydrates degradation. The enrichment of genes encoding CAZymes involved in different types of enzymes revealed the higher capability of degrading diverse and complex substrates in the rumen of dairy buffaloes. The GHs are common enzymes in rumen that catalyze the hydrolysis of glycosidic bond in complex carbohydrates, which are involved in deconstructing plant biomass such as cellulose, hemicellulose, and starch [52]. The large amount of significantly enriched GHs identified in the rumen microbiome of dairy buffaloes suggests the strong fiber degradation roles in the rumen of dairy buffaloes. The extremely more abundant GHs families (GH68, GH70, GH71) with over 50-fold enrichment revealed higher ability of acting on sucrose, maltodextrins/starch, and energy production in dairy buffaloes [53, 54]. It has been suggested that utilizing microbial enzymes belonging to AA family could help degrade the non-carbohydrate structural components (e.g. lignin) since the AAs contain a group of ligninolytic enzymes or multi-copper oxidases [55]. The AA1 family plays important roles in lignin degradation by acting as laccase [56]. The AA1 family-encoded genes only identified in the rumen of dairy buffaloes with a relative high abundance (CPM = 185) indicates that dairy buffaloes might be more capable or more efficient in degrading the recalcitrant lignin, which contribute to the better adaptability to less-digestible forage.

Liu et al. investigated the fecal resistome of dairy cattle and identified 329 ARGs that putatively conferred resistance to 17 classes of antimicrobials [5]. Recent studies reported that the rumen microbiome carries abundant ARGs, which may be transmitted to humans via environment (i.e. soil and water) through saliva or the flow of rumen microbial biomass to the gut [11]. In this study, more than 50% of ARGs and resistant antimicrobials classes were detected in the rumen of dairy cows and buffaloes, suggesting the necessity to pay attention to the ruminal resistome, especially for those that confer resistance to clinically important antibiotics. *Mcr-1* is a plasmid-mediated colistin resistance gene that was firstly discovered in the pork, chicken and human in China, and then identified globally [57], which compromise last-line treatment options for human health especially for the multidrug-resistant Gram-negative pathogen infections [58]. It is suggested that the spread of *mcr-1* is from livestock sector to human beings [2]. The high *mcr-1* positive detection rate in the rumen of dairy cows (81%) and dairy buffaloes (100%) indicates the *mcr-1* widespread in dairy cows and buffaloes in the south of China, thus the use of colistin in dairy industry should be reconsidered. Linezolid is one of the last antimicrobial treatment options in human clinical medicine and has not been approved for applying in livestock industry [6]. However, several linezolid resistant genes (*cfrA*, *cipA*, *clbA*, *clbB*, *clbC*) with high relative abundance (average CPM > 600 for each gene) were identified in the rumen samples of dairy cows and dairy buffaloes, and they also confirmed in other studies [59, 60]. The high prevalence of ARGs that conferred resistance to clinical-important antibiotics in the rumen microbiota should be taken into account to address potential concerns for public health. The microbial genomes contain functional integrative conjugative elements that determine the possibility of transferring ARGs into human pathogens or zoonotic pathogens [13]. Further work is needed to investigate these aspects.

It has been reported that diets [5], ages [61], and living environment [12] play important roles in shaping gut resistome in animals. The distinct resistome between dairy cows and buffaloes indentified in this study may be attributed to the combined effects of distinct diets, ages, environmental conditions and host genetics. Among them, the diet of dairy buffaloes hade lower nutritional level (e.g., CP) and less abundant ingredient types. It’s noted that 60% corn silage was utilized in the dairy buffaloes’ diet while only 16.7% used in the dairy cows’ diet (Table S1), which may contribute the more diverse resistome in the rumen of dairy buffaloes. The improved adaptability to less-digestible roughage in dairy buffaloes [16] may harbor varied microbiota with diverse ARGs. In addition, the close interactions between dairy buffaloes and waterlogged conditions [19] increase the ARGs horizontal transfer chances, leading to a more abundant resistome. Interestingly, the 18 unique ARGs were detected in each ruminal sample of dairy buffaloes, but they were absent in any ruminal sample from dairy cows. This is also confirmed in another metagenomic dataset (unpublished) of dairy cows. This may be attributed to the distinct rumen microbiome between two host species since microbial resistome are mainly structured by the bacterial phylogeny [8]. These 18 unique ARGs have not been detected in the ruminal resistome of different beef cattle species with varied diets in a previous study [11]. In another study, Cao et al. collected gut samples from 10 different migratory bird species (total number = 99) and characterized their microbiome and resistome using metagenomics [62]. Four dairy buffalo unique ARGs (*adeA*, *sul3*, *mexC*, *tet41*) were identified in this dataset, but the detection rate was only 1–14% and none of them was distributed in every sample of one certain species. When compared with the study on the swine farm related samples (human feces, pig feces, soil, sewage, and ventilation dust) [63], three (*tet34*, *sul3*, *iri*) buffalo unique ARGs were also identified. Based on the above comparison with more diverse gut microbiome, most of unique ARGs in dairy buffaloes were conserved, indicating the uniqueness of ruminal resistome and ARGs profiles.

Among the unique ARGs, *tcmA* was highlighted since it showed greatest positive correlation with a gene module consisting of 93 co-expressed ARGs. The *tcmA* is predicted to confer resistance to tetracenomycin C via an active efflux system [64]. Tetracenomycin C is a narrow spectrum anthracycline antibiotic, which is active against Gram-positive bacteria and also shows antitumor effect [65]. In this study, we identified more than 40 bacteria under *Lactobacillus* genus that were highly related to *tcmA,* suggesting that *tcmA* might be widely harbored and/or more likely horizontally transferred in *Lactobacillus* bacteria. Among them, *L. amylovorus* and *L. acidophilus* showed strongest association with *tcmA*, which is supported by the taxonomic alignment using ARGs-containing contigs. The lactic bacteria *Lactobacillus amylovorus* was firstly isolated from waste-corn fermentations in cattle, and can secret starch-hydrolyzing enzyme (a-amylase) and rapidly digest cornstarch [66]. It has been found that *L. amylovorus* and *L. acidophilus* exert beneficial effects on gastrointestinal immunomodulation and health as candidate bacterial therapeutics, such as mitigating the bovine respiratory pathogens in dairy calves [67]. The probiotic properties of *L. amylovorus* and *L. acidophilus* have been well studied [68, 69], suggesting the promising cholesterol-lowering properties [70], anti-pathogens effect [71], and prevention of disease in human and dairy calves [72]. The significantly higher relative abundance of these two species especially for *L. amylovorus* (2% in young dairy buffaloes) in the rumen of dairy buffaloes may provide probiotic resource for fermented feed. The detected *tcmA* will provide fundamental knowledge and potential strategies to reduce ARG transmission risk when isolated *Lactobacillus* probiotic from the rumen of dairy buffaloes.

## Conclusions

Ruminal bacterial composition varied significantly between dairy cows and dairy buffaloes. The majority of differed KEGG pathways and CAZymes were enriched in the dairy buffaloes with GH68, GH70, GH71, GT48 and AA1were extremely significantly higher than dairy cows. Dairy buffaloes showed distinct resistome profiles and clusters from dairy cows. A total of 18 ARGs conferring resistance to 16 antibiotic classes were uniquely detected in the dairy buffaloes. *tcmA* plays a central role in the ruminal resistome and is highly associated with *L. amylovorus* and *L. acidophilus*. Our results provide novel insights into the microbiome and resistome of dairy buffaloes, the identified unique ARGs and associated bacteria will help develop strategies to prevent the transmission of ARGs.

## Supplementary Information

**Additional file 1: Table S1.** The diet ingredents of dairy cows and dairy buffaloes. **Table S2.** The relative abundance of bacteria at domain level in individuals. **Table S3.** The significantly different CAZymes encoded genes identified in the rumen microbiota between dairy cows and buffaloes. **Table S4**. ARGs and their abundance in dairy cows and dairy buffaloes. **Table S5.** ARGs and their AMR phenotypes in the dairy buffaloes. **Table S6.** The relative abundance of unique ARGs in different age groups of dairy buffaloes.

## Data Availability

The metagenomics raw sequences of rumen samples were submitted into the NCBI Sequence Read Archive (SRA) under the accession numbers PRJNA718720, other relevant data are available upon request.

## Abbreviations

AAs: Auxiliary activities
AMR: Antimicrobial resistance
ARGs: Antimicrobial resistance genes
BWA: Burrows-Wheeler alignment
CARD: Comprehensive Antimicrobial Resistance Database
CAZy: Carbohydrate-Active enZYmes Database
CBMs: Carbohydrate-binding modules
CPM: Counts per million reads
FDR: False discovery rate
FC: Fold change
GHs: Glycoside hydrolases
GTs: Glycosyltransferase
KEGG: Kyoto Encyclopedia of Genes and Genomes database
LDA: Least discriminant analysis
LEfSe: LDA effect size
NR: Non-redundant
PCoA: Principal-coordinate analysis
PLs: Polysaccharide lyases
PLS-DA: Partial least-squares discrimination analysis
SDFs: Significantly different features
WGCNA: Weighted gene co-expression network analysis
